# Risk Factors for Depressive Status in Patients With Obsessive-Compulsive Disorder: A Case-Control Study

**DOI:** 10.62641/aep.2264

**Published:** 2026-10-15

**Authors:** Tian Tang, Ying He, Cong Tang, Xiaorong Yang

**Affiliations:** ^1^Second Outpatient Department, West China Hospital, Sichuan University, 610041 Chengdu, Sichuan, China; ^2^Psychological Health Center, West China Hospital, Sichuan University, 610041 Chengdu, Sichuan, China; ^3^Department of Rehabilitation Medicine, Chengdu Eighth People's Hospital, 610081 Chengdu, Sichuan, China; ^4^Outpatient Department, West China Hospital, Sichuan University, 610041 Chengdu, Sichuan, China

**Keywords:** obsessive-compulsive disorder, comorbidity, depression, clinical characteristics, LASSO regression

## Abstract

**Background:**

This study aimed to characterize clinical features of patients with obsessive-compulsive disorder (OCD) comorbid with depression, and to explore the relationship between OCD symptom severity and Hamilton Depression Rating Scale (HAMD) scores.

**Methods:**

This retrospective study enrolled 202 patients with OCD treated at West China Hospital of Sichuan University between January 2021 and December 2025, none of whom had received anti-OCD or antidepressant pharmacotherapy during the 8 weeks prior to enrolment. Of these participants, 96 patients presented comorbid depression (depression group, HAMD-17 ≥8), and 106 patients had no comorbid depression (non-depression group, HAMD-17 < 8). Least Absolute Shrinkage and Selection Operator (LASSO) regression was used to identify independent variables associated with comorbid depression for constructing a Logistic regression model. Pearson correlation analysis was performed to assess the relationship between Yale–Brown Obsessive-Compulsive Scale (Y-BOCS) total score, obsession score and compulsion score with HAMD-17 scores in the depression group.

**Results:**

The depression group showed a significantly longer disease course (98.62 ± 9.00 months vs. 93.02 ± 8.96 months, *t* = 4.427, *p* < 0.001) and a lower proportion of first-time consultations (32.29% vs. 49.06%, χ^2^ = 5.849, *p* = 0.016). Comorbid anxiety (28.12% vs. 8.49%, χ^2^ = 13.260, *p* < 0.001) and family history of psychosis (23.96% vs. 5.66%, χ^2^ = 13.718, *p* < 0.001) were more frequent in the depression group. LASSO regression (1SE lambda = 0.017) selected six variables for the logistic model: comorbid anxiety, family history of psychosis, medical payment type, OCD severity (moderate-severe vs. mild), obsession severity and compulsion severity. Multivariate logistic regression analysis identified comorbid anxiety (odds ratio (OR) = 3.418, 95% confidence interval (CI): 1.202–9.715), family history of psychosis (OR = 2.649, 95% CI: 1.050–6.680), OCD severity (OR = 1.618, 95% CI: 1.035–2.529), obsession severity (OR = 1.826, 95% CI: 1.059–3.148), and compulsion severity (OR = 2.980, 95% CI: 1.361–6.527) as independent risk factors for comorbid depression (all *p* < 0.05). Pearson correlation analysis revealed that Y-BOCS total score, obsession score, and compulsion score were all significantly positively correlated with HAMD score (r = 0.577, r = 0.649, r = 0.721, respectively; all *p* < 0.05).

**Conclusions:**

Comorbid anxiety, a family history of psychosis, and greater severity of OCD may significantly increase the risk of comorbid depression in patients with OCD. The severity of obsessive-compulsive symptoms demonstrates a moderate to strong positive correlation with depression severity.

## Introduction

Obsessive-compulsive disorder (OCD) is characterized by intrusive obsessive thoughts and repetitive compulsive behaviours. With a global lifetime prevalence of approximately 1%–3%, OCD symptoms often consume significant time, causing persistent psychological distress and functional impairment [1]. In clinical practice, depressive symptoms are among the most common and disruptive comorbidities of OCD. Patients with comorbid depression tend to present with higher obsessive-compulsive symptom burden, anxiety levels and functional impairment. Identifying and managing this comorbidity is directly relevant to treatment selection and prognosis [2]. Existing research has typically examined comorbidity rates, clinical burden, symptom dimension relationships and related influencing factors, covering aspects such as obsessive-compulsive symptom severity, differences between obsessions and compulsions, family genetic background, comorbid anxiety and functional impairment. However, gaps remain in variable selection methodology, symptom dimension stratification, and the construction of comorbid depression risk models. In particular, evidence integrating obsessions, compulsions, family history of psychosis, and comorbid anxiety into a single analytical framework remains scarce [3, 4, 5]. Based on the clinical evidence, the present study adopted a retrospective case-control design to compare clinical characteristics of patients with OCD with or without comorbid depression, to apply Least Absolute Shrinkage and Selection Operator (LASSO) regression to identify variables for logistic regression model construction, and to explore the correlation between Yale–Brown Obsessive-Compulsive Scale (Y-BOCS) scores and Hamilton Depression Rating Scale (HAMD) scores. This study hypothesized that comorbid anxiety, family history of psychosis, and greater severity of obsessive-compulsive symptoms are associated with an increased risk of comorbid depression in patients with OCD, and that a higher obsessive-compulsive symptom burden is associated with higher depressive symptom scores.

## Materials and Methods

### General Information

Using a retrospective study design, the sample size was calculated according to the formula for a group-based case-control study, with significance level α = 0.05 (two-sided, Z_α/2_ = 1.96), power (1–β) = 0.90 (Z_β_ = 1.282), estimated non-depression group exposure rate = 0.20, case group exposure rate derived from an OR of 2.76 from the literature, yielding a minimum required sample of approximately 190. Considering the accuracy of clinical data and potential missing data, a total of 202 patients with OCD were included after reviewing 336 medical records at West China Hospital of Sichuan University from January 2021 to December 2025. Of the 336 records reviewed, 134 patients were excluded based on the exclusion criteria or due to incomplete data, resulting in 202 eligible cases (Fig. 1). Among them, 96 patients had comorbid depression (depression group, diagnosed according to the Diagnostic and Statistical Manual of Mental Disorders, Fifth Edition [DSM-5] [6] criteria, HAMD-17 ≥8 [7]), and the other 106 patients did not have comorbid depression (non-depression group, HAMD-17 < 8).

**Fig. 1.**
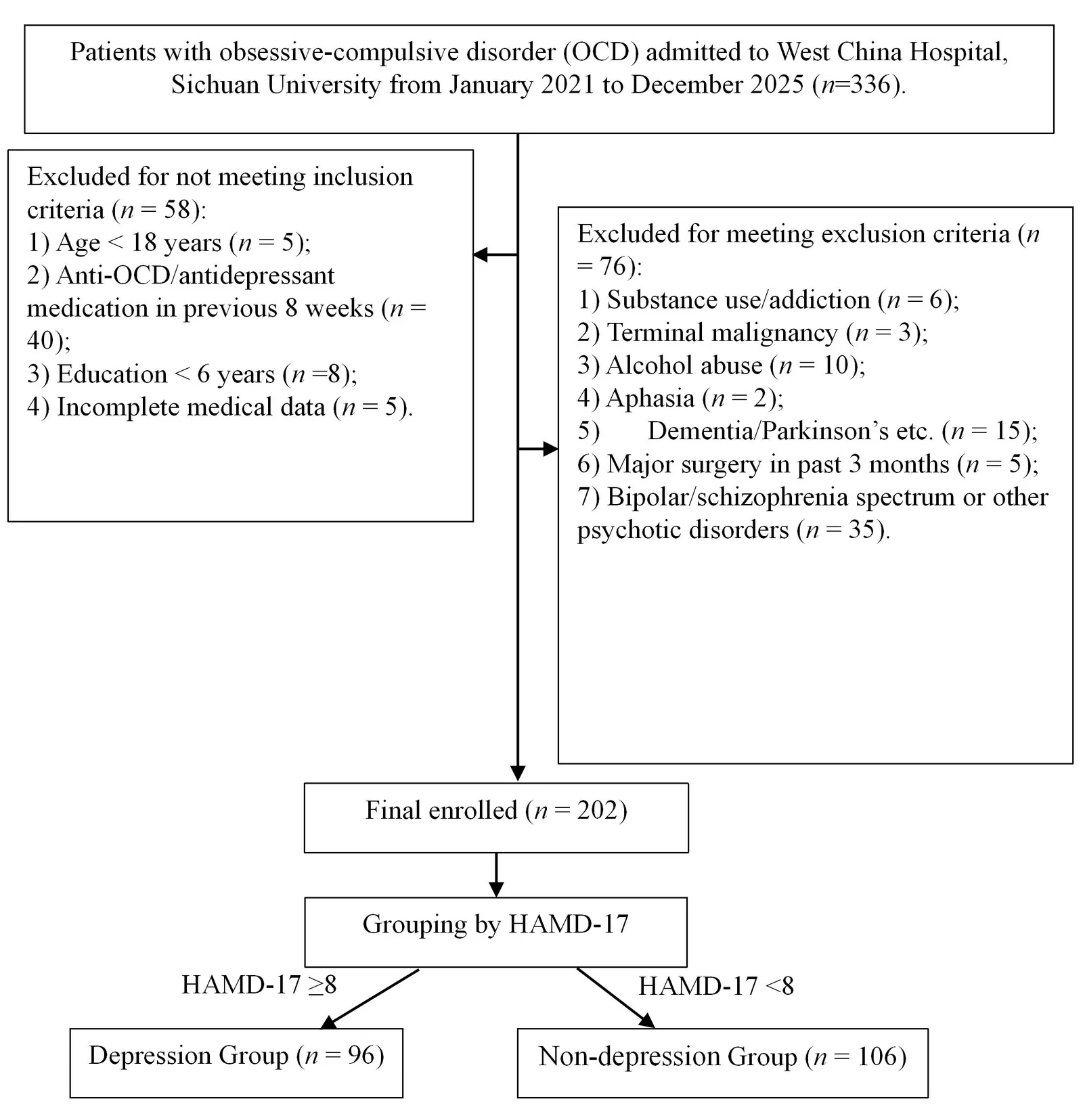
**Flowchart of patient enrollment and group allocation**. HAMD, Hamilton Depression Rating Scale; OCD, obsessive-compulsive disorder.

The diagnostic criteria were as follows: (1) The diagnostic criteria for OCD referenced the DSM-5, and patients had a Y-BOCS score ≥16 [8], with the presence of obsessions, compulsions, or both. (2) The diagnostic criteria for depression referenced the DSM-5, and patients were required to have HAMD-17 [9] score ≥8 (indicating at least mild depression). A cutoff of ≥8 was adopted because it is widely used in clinical research to define mild depression, enabling the inclusion of early depressive symptoms while maintaining clinical relevance.

The inclusion criteria were as follows: (1) aged ≥18 years; (2) no anti-OCD or antidepressant medications use within the preceding 8 weeks; (3) ≥6 years of formal education (to avoid compromised scale validity stemming from limited literacy or cultural barriers); and (4) complete patient data available.

The exclusion criteria were as follows: (1) illicit drugs or substance dependence; (2) diagnosis of terminal malignant tumours; (3) alcohol abuse; (4) aphasia; (5) diagnosis of senile dementia, Parkinson’s disease; (6) major surgery within the prior 3 months; or (7) prior diagnosis of bipolar disorder, schizophrenia spectrum disorder, or other psychotic disorders. Patients with other anxiety spectrum disorders (e.g., panic disorder, social anxiety disorder) were not excluded; relevant comorbidities were documented as the variable “comorbid anxiety”.

### Collection of General Information

Based on medical record systems and scale assessment records, patient baseline data were extracted, including age, body mass index (BMI), disease course, sex, education level, marital status, consultation history, household registration type, comorbid physical diseases, comorbid anxiety, monthly family income, family history of psychosis, medical payment type, OCD severity (classified as mild vs. moderate-severe), obsession dimension severity (mild vs. moderate-severe), compulsion dimension severity (mild vs. moderate-severe), and precipitating factors.

### Y-BOCS Assessment

Y-BOCS [10] assessment records completed within 48 hours of admission were retrieved from the medical records to determine the severity of obsessive-compulsive symptoms. The scale assessment was independently conducted by two attending physicians or above who had received standardized training and had clinical psychiatric evaluation experience. Before the assessment, standardized scoring criteria training was conducted, and any items with disagreements were discussed to reach a final consensus record. Prior to the study, 30 scale records were randomly selected for consistency testing, yielding an intraclass correlation coefficient (ICC) of 0.86 for total Y-BOCS score, indicating good interrater agreement. The Y-BOCS includes two dimensions: obsessions and compulsions. Each dimension contains 5 items, each scored on a 5-point scale from 0 to 4. Higher scores indicate more severe OCD symptoms, with a maximum of 20 points per dimension and a total maximum score of 40. Each dimension score range denotes distinct symptom severity: 0–7 for subclinical symptoms, 8–15 for mild symptoms, 16–23 for moderate symptoms, 24–31 for severe symptoms, and 32–40 for extreme OCD symptoms [10, 11].

### HAMD Assessment

HAMD-17 assessment records completed within 48 hours of admission, concurrently with the Y-BOCS assessment, were retrieved to determine the severity of depressive symptoms. The HAMD-17 was independently rated by two attending physicians or above who had received standardized training and had psychiatric scale evaluation experience. For cases with large rating discrepancies, a third senior physician reviewed the scores. Prior to the study, 30 scale records were randomly selected for consistency testing, yielding an ICC of 0.88 for total HAMD-17 score, indicating good interrater agreement. HAMD-17, a modified version, encompasses five symptom domains: emotional/cognitive symptoms, psychomotor symptoms, anxiety-related symptoms, sleep disturbances and somatic symptoms, and consists of 17 individual rating items [12]. The total score ranges from 0 to 52, with a score ≥8 suggesting possible mild depression [7].

### Statistical Methods

All study data were analysed using SPSS version 23.0 (IBM Corp., Armonk, NY, USA) and R version 4.0.0 (R Foundation for Statistical Computing, Vienna, Austria). To minimize information bias, all scale assessments were conducted by trained raters using standardized procedures, and inter-rater reliability was confirmed with ICCs > 0.85. Continuous variables, all confirmed to follow a normal distribution by the Kolmogorov–Smirnov test, were expressed as mean ± standard deviation. Comparisons between groups for such data were performed using the independent samples *t*-test. Count data, such as medical history, were described using frequencies and percentages (%), and comparisons between groups were made using the chi-square test ($\chi^{2}$). To address confounding bias, LASSO regression was used for variable selection, which shrinks redundant coefficients and mitigates overfitting, thereby yielding a more parsimonious and interpretable model. Additionally, the final logistic regression model included all selected variables to mutually adjust for potential confounding. Given the risks of model overfitting and multicollinearity among independent variables, LASSO regression was chosen in preference to stepwise approaches, as it applies regularization that shrinks coefficients of redundant variables to zero and improving model stability and interpretability. The 1SE lambda model was selected according to the Akaike Information Criterion (AIC) and Bayesian Information Criterion (BIC), and the variables entering the model at this lambda were used for subsequent multivariate analysis. A *p*-value < 0.05 was considered statistically significant in univariate analyses, and variables meeting this criterion were advanced to LASSO regression. Multivariate analysis of factors associated with the risk of comorbid depression was conducted using unconditional logistic regression with the enter method for independent variables. Correlation analysis was performed using Pearson’s method. A two-sided *p*-value < 0.05 was considered statistically significant.

## Results

### Univariate Analysis of Depression and Non-Depression Groups

Univariate comparison of clinical characteristics between the depression and non-depression groups demonstrated that patients with depression had a significantly longer disease course (98.62 ± 9.00 months vs. 93.02 ± 8.96 months, *t* = 4.427, *p* < 0.001) and a significantly lower proportion of first-time consultations (32.29% vs. 49.06%, $\chi^{2}$ = 5.849, *p* = 0.016). In terms of clinical features, the depression group had significantly higher proportions of comorbid anxiety (28.12% vs. 8.49%, $\chi^{2}$ = 13.260, *p* < 0.001) and a family history of psychosis (23.96% vs. 5.66%, $\chi^{2}$ = 13.718, *p* < 0.001). Additionally, a higher proportion of patients in the depression group paid medical expenses out-of-pocket (11.46% vs. 2.83%, $\chi^{2}$ = 5.814, *p* = 0.016). Disease severity assessment showed that the depression group had significantly higher proportions of patients classified as having moderate-severe OCD (66.67% vs. 51.89%, $\chi^{2}$ = 4.564, *p* = 0.033), moderate-severe obsessions (61.46% vs. 44.34%, $\chi^{2}$ = 5.920, *p* = 0.015), and moderate-severe compulsions (63.54% vs. 45.28%, $\chi^{2}$ = 6.764, *p* = 0.009). No statistically significant differences were found between the two groups in age, BMI, sex, education level, marital status, household registration type, comorbid physical diseases, monthly family income, or precipitating factors (all *p* > 0.05, Table 1).

**Table 1.** **Univariate analysis results for depression and non-depression groups**.

Items		Depression group (n = 96)	Non-depression group (n = 106)	*t*/$\chi^{2}$	*p*
Age (years)		39.61 ± 7.21	37.84 ± 7.95	1.651	0.100
BMI (kg/m^2^)		23.77 ± 2.04	23.82 ± 2.11	–0.171	0.865
Disease course (months)		98.62 ± 9.00	93.02 ± 8.96	4.427	<0.001
HAMD score		23.97 ± 4.21	5.11 ± 1.40	43.550	<0.001
Gender [n, (%)]				1.217	0.270
	Male	59 (61.46)	57 (53.77)		
	Female	37 (38.54)	49 (46.23)		
Education level [n, (%)]				2.961	0.085
	High school or below	69 (71.88)	64 (60.38)		
	College or above	27 (28.13)	42 (39.62)		
Marital status [n, (%)]				1.781	0.182
	Married	81 (84.37)	96 (90.57)		
	Unmarried/Divorced	15 (15.63)	10 (9.43)		
Consultation history [n, (%)]				5.849	0.016
	First visit	31 (32.29)	52 (49.06)		
	Multiple visits	65 (67.71)	54 (50.94)		
Household registration [n, (%)]				1.294	0.255
	Rural	23 (23.96)	33 (31.13)		
	Urban	73 (76.04)	73 (68.87)		
Comorbid physical disease [n, (%)]				1.310	0.252
	Yes	14 (14.58)	22 (20.75)		
	No	82 (85.42)	84 (79.25)		
Comorbid anxiety [n, (%)]				13.260	<0.001
	Yes	27 (28.12)	9 (8.49)		
	No	69 (71.88)	97 (91.51)		
Monthly family income [n, (%)]				3.453	0.063
	<6000 CNY	43 (44.79)	34 (32.08)		
	≥6000 CNY	53 (55.21)	72 (67.92)		
Family history of psychosis [n, (%)]				13.718	<0.001
	Yes	23 (23.96)	6 (5.66)		
	No	73 (76.04)	100 (94.34)		
Medical payment type [n, (%)]				5.814	0.016
	Out-of-pocket	11 (11.46)	3 (2.83)		
	Insurance payment	85 (88.54)	103 (97.17)		
OCD severity [n, (%)]				4.564	0.033
	Mild	32 (33.33)	51 (48.11)		
	Moderate-severe	64 (66.67)	55 (51.89)		
Obsession severity [n, (%)]				5.920	0.015
	Mild	37 (38.54)	59 (55.66)		
	Moderate-severe	59 (61.46)	47 (44.34)		
Compulsion severity [n, (%)]				6.764	0.009
	Mild	35 (36.46)	58 (54.72)		
	Moderate-severe	61 (63.54)	48 (45.28)		
Y-BOCS score		27.91 ± 4.88	22.08 ± 3.50	9.824	<0.001
Precipitating factors [n, (%)]				1.112	0.292
	Yes	71 (73.96)	85 (80.19)		
	No	25 (26.04)	21 (19.81)		

“Multiple visits” was defined as ≥3 consultations for OCD-related or depression-related reasons within one year. “Comorbid physical diseases” included hypertension, diabetes mellitus, coronary heart disease, etc. “Family history of psychosis” refers to a confirmed diagnosis of depression, anxiety disorder, schizophrenia, or other psychotic disorders in first-degree relatives. Comorbid anxiety was defined as a Hamilton Anxiety Rating Scale (HAMA) score ≥8. Values in parentheses are percentages (%). BMI, body mass index; HAMD, Hamilton Depression Rating Scale; Y-BOCS, Yale-Brown Obsessive-Compulsive Scale; OCD, obsessive-compulsive disorder. 1 CNY ≈ 0.147 USD.

### LASSO Analysis for Variable Selection

LASSO analysis was performed to the eight univariately significant clinical indicators: disease course, consultation history, comorbid anxiety, family history of psychosis, medical payment type, OCD severity, obsession severity and compulsion severity. The 1SE-optimized model (lambda = 0.017) had an AIC of 192.5 and BIC of 215.7. The 1SE lambda (0.017, 1SE criterion) was prioritized based on AIC and BIC performance metrics, retaining six independent variables: comorbid anxiety, family history of psychosis, medical payment type, OCD severity, obsession severity, and compulsion severity. The cross-validation curve for variable selection is shown in Fig. 2, and the coefficient path plot is shown in Fig. 3.

**Fig. 2.**
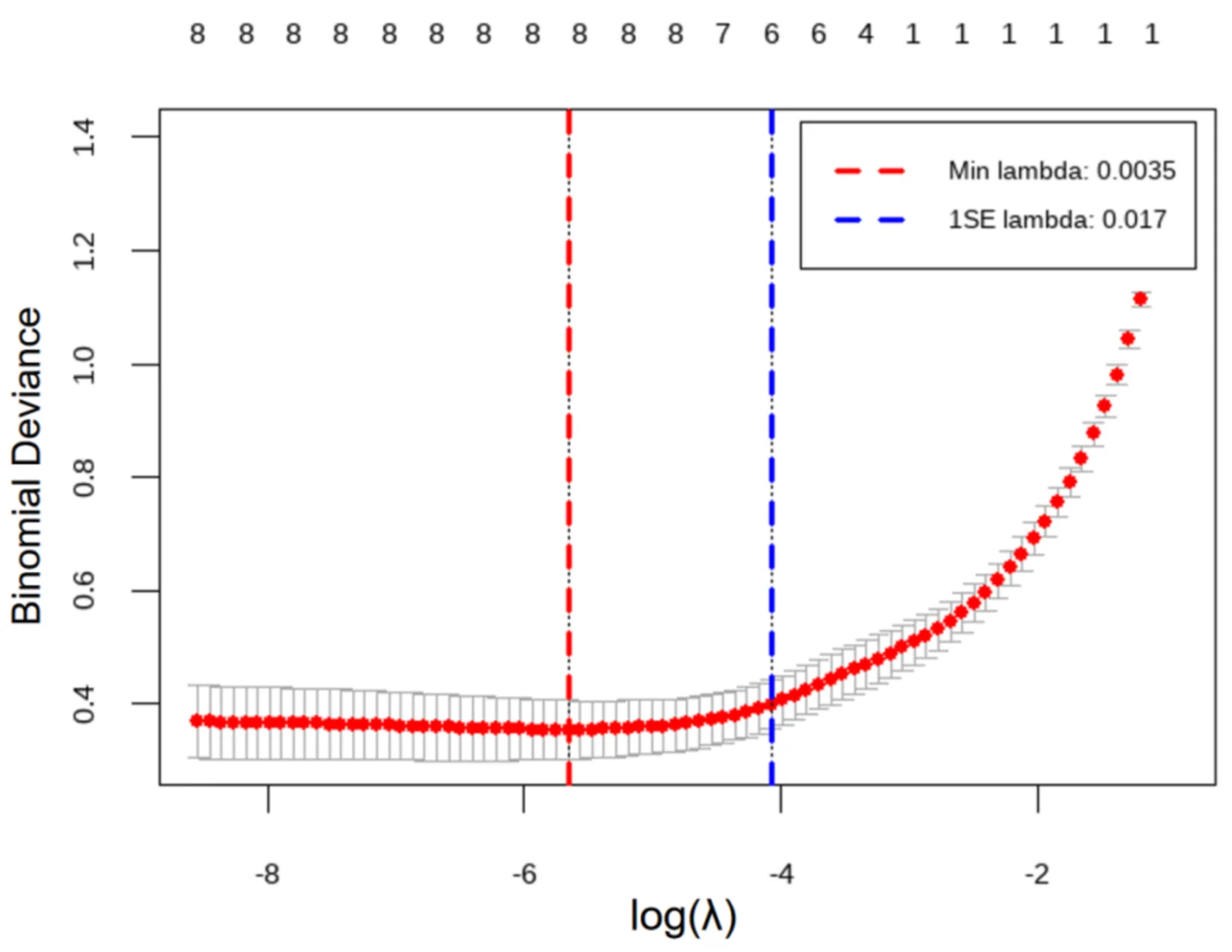
**Cross-validation curve of LASSO regression for screening risk factors of comorbid depression in OCD patients**.

**Fig. 3.**
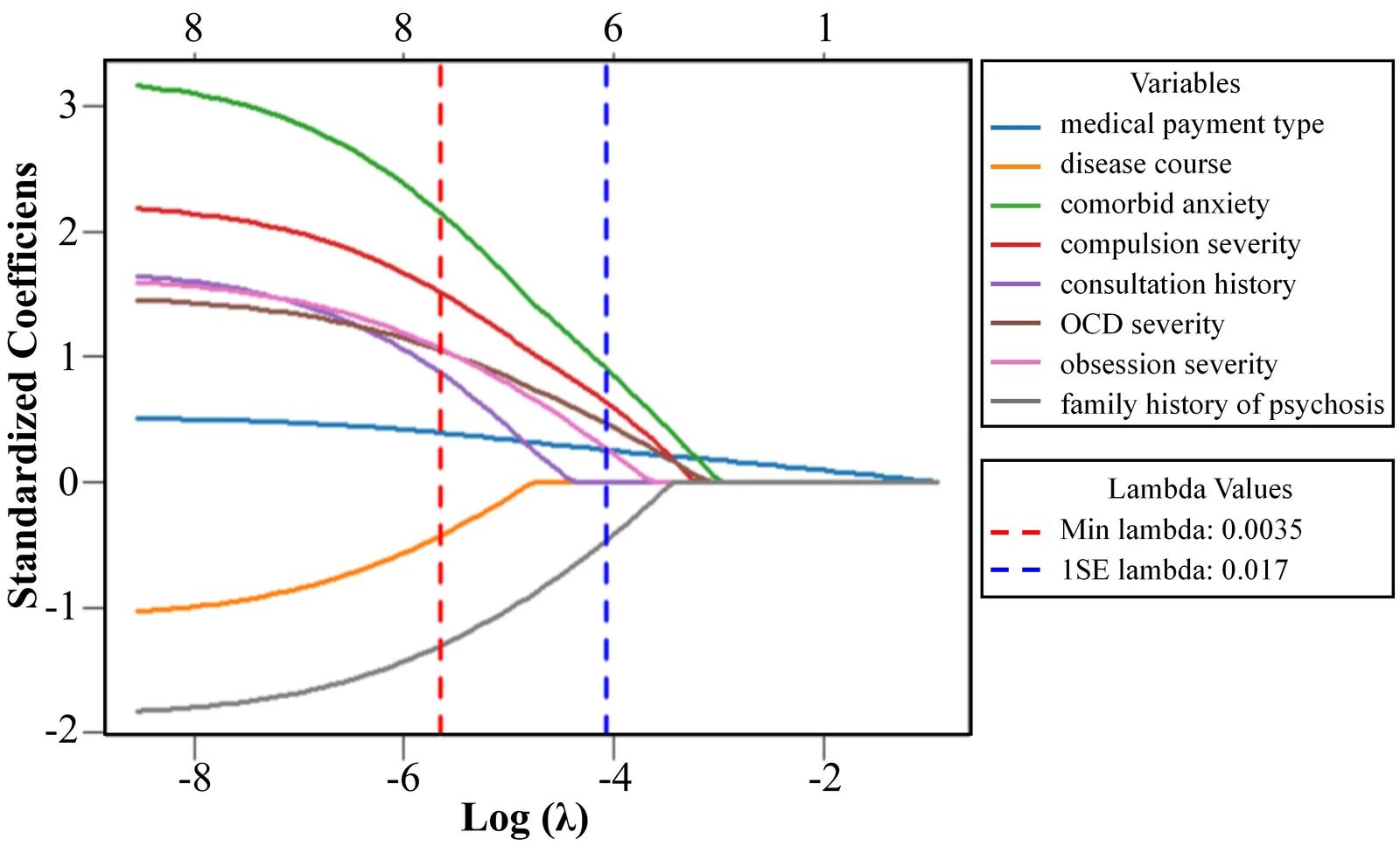
**Coefficient paths of variables selected by LASSO regression**. OCD, obsessive-compulsive disorder.

### Correlation Between OCD Severity and HAMD-17 Scores

Simple linear correlation analysis, including scatter plots and calculation of the correlation coefficient, revealed a significant positive correlation between total Y-BOCS score and HAMD score, as well as obsession score and HAMD score, and compulsion score and HAMD score (r = 0.577, r = 0.649, r = 0.721, respectively; all *p* < 0.05, Fig. 4).

**Fig. 4.**
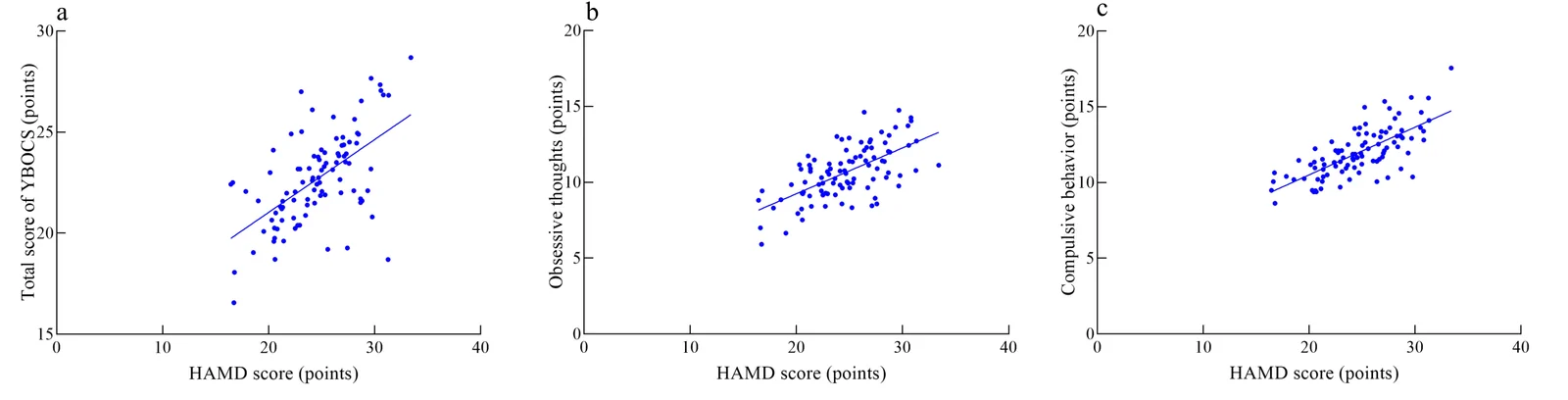
**Scatter plots showing correlations of (a) Y-BOCS total score, (b) obsessive thoughts score, and (c) compulsive behavior score with HAMD score in the depression group**. HAMD, Hamilton Depression Rating Scale; Y-BOCS, Yale-Brown Obsessive-Compulsive Scale.

### Analysis of Risk Factors for Comorbid Depression in Patients With OCD

Based on the LASSO regression results, an unconditional logistic regression model was constructed using comorbid anxiety (no = 0, yes = 1), family history of psychosis (no = 0, yes = 1), medical payment type (insurance = 0, out-of-pocket = 1), OCD severity (mild = 0, moderate-severe = 1), obsession severity (mild = 0, moderate-severe = 1), and compulsion severity (mild = 0, moderate-severe = 1). The enter method was used for variable entry. The results showed that comorbid anxiety (odds ratio [OR] = 3.418, 95% confidence interval [CI]: 1.202–9.715), family history of psychosis (OR = 2.649, 95%CI: 1.050–6.680), OCD severity (OR = 1.618, 95% CI: 1.035–2.529), obsession severity (OR = 1.826, 95% CI: 1.059–3.148), and compulsion severity (OR = 2.980, 95% CI: 1.361–6.527) were risk factors for comorbid depression in patients with OCD (all *p* < 0.05). Medical payment type was not statistically significant (*p* = 0.095, Table 2).

**Table 2.** **Analysis of risk factors for comorbid depression in OCD patients**.

Indicator	β	SE	Wald $\chi^{2}$	*p*	OR	95%CI
Comorbid anxiety	1.229	0.533	5.317	0.018	3.418	1.202–9.715
Family history of psychosis	0.974	0.472	4.258	0.047	2.649	1.050–6.680
Medical payment type	0.733	0.397	3.409	0.095	2.081	0.956–4.532
OCD severity	0.481	0.228	4.451	0.045	1.618	1.035–2.529
Obsession severity	0.602	0.278	4.689	0.043	1.826	1.059–3.148
Compulsion severity	1.092	0.400	7.453	<0.001	2.980	1.361–6.527
Constant	1.402	0.772	3.298	0.087	-	-

SE, standard error; OR, odds ratio; CI, confidence interval; OCD, obsessive-compulsive disorder.

## Discussion

The core manifestations of OCD involve intrusive obsessions and repetitive compulsions. Compulsions are often performed to relieve the distress caused by obsessions or to prevent feared consequences. During this process, individuals may form a negative reinforcement cycle of ritualistic behaviours to reduce internal anxiety, and symptom recurrence can further accumulate psychological and social functional burden over time [1, 13]. These findings indicate that the depression group had a longer disease course and a lower proportion of first-time consultations, suggesting that a chronic, fluctuating course of obsessive-compulsive symptoms and repeated medical visits may be associated with depressive phenotype formation. Prolonged symptom duration can increase emotional exhaustion, helplessness, and cumulative functional impairment, making patients more prone to developing depressive phenotypes amid long-term, high-load internal conflict. Multiple consultations may also be related to poor treatment response, treatment-refractory illness, and cumulative functional impairment, thereby contributing to sustained stress exposure, feelings of failure in coping, and depressive symptom maintenance [13, 14]. However, in the LASSO selection, disease course and consultation history did not enter the final model, suggesting that their effect on comorbid depression may be partially mediated by more proximal clinical variables such as obsessive-compulsive symptom severity and comorbid anxiety, rather than acting as independent risk factors [15, 16]. Clinically, this finding suggests that for patients with chronic OCD and repeated medical visits, merely controlling symptoms may be insufficient; a simultaneous assessment of emotional state and functional impairment is needed to mitigate the persistent risk of depressive phenotypes.

Research has pointed out the high degree of coupling between anxiety and OCD at the mechanistic level of emotional arousal, threat monitoring and avoidance behaviours, with anxiety often serving as a proximal driver of OCD symptom exacerbation [13]. These findings indicate that moderate-severe OCD severity is a risk factor for comorbid depression in patients with OCD (OR = 1.618, 95% CI: 1.035–2.529), meaning that after controlling for other variables, the odds of comorbid depression in moderate-severe patients were approximately 1.618 times those of mild patients. This finding suggests that when obsessive-compulsive symptom burden reaches a moderate-severe level, the risk of depression may show a clinically identifiable increase. A possible mechanism is that more severe OCD keeps individuals in a chronic state of hypervigilance, directing attentional resources more toward threat cues and uncertain information, which triggers repeated checking, washing and other compulsive behaviours for temporary relief [17, 18]. When compulsions fail to provide a stable sense of control, individuals are more likely to experience core depressive experiences such as low mood, loss of interest, and hopelessness. In this study, Y-BOCS, obsession, and compulsion scores exhibited significant positive correlations with HAMD-17 depressive symptom severity scores (r = 0.577, 0.649, 0.721), with compulsion score showing the highest correlation. This finding implies that the time consumption, disruption of life rhythm, and social functional limitations stemming from overt compulsive behaviours may constitute key clinical mechanisms linking exacerbations of obsessive-compulsive symptomatology to worsening depressive manifestations. Other studies have shown that anxiety and depression also share common pathways at the neurobiological level, including stress axis activation, inflammatory responses and impaired reward processing [19, 20]. The chronicity of the anxious state can promote the formation of depressive phenotypes through physiological load accumulation and sleep disturbances [21]. A study by Belli *et al*. [22] based on a 6-year prospective follow-up of 324 patients with COD found that OCD symptoms predicted depression severity in the following year, but depressive symptoms could not inversely predict OCD symptoms. Although the present study is a retrospective case-control design and cannot verify temporal sequence, the depression group in this study had a longer disease course, more severe OCD, and a positive correlation between total Y-BOCS score and HAMD score (r = 0.577), which is directionally consistent with their conclusion that “obsessive-compulsive symptom burden is associated with greater depression”. The difference lies in the fact that this study further stratified obsessions and compulsions, revealing that the compulsion score had the highest correlation with HAMD score (r = 0.721), suggesting that the functional impairment caused by the behavioural dimension may reflect depressive burden more effectively than total score alone. Hathway *et al*. [23] further found in 243 patients with OCD that those with comorbid depression scored significantly higher on all OCD symptom subtypes, obsession and compulsion severity, perfectionism, and disgust sensitivity and propensity, with OCD severity being independently associated with depressive symptoms. These findings are consistent with this, suggesting that OCD severity is a key indicator in the identification of comorbid depression. The novelty of this study involves implementing LASSO regression for preliminary variable screening prior to constructing logistic model. This method effectively mitigates of multicollinearity. It further indicates that comorbid anxiety (OR = 3.418) and a family history of psychosis (OR = 2.649) are associated with comorbid depression. These observations suggest that evaluating only OCD symptom severity may be insufficient and that comprehensive risk stratification incorporating anxiety status and family history is warranted.

This study also found that a family history of psychosis was associated with the risk of comorbid depression (OR = 2.649, 95% CI: 1.050–6.680). A family history of psychosis often suggests a higher transdiagnostic individual vulnerability to psychiatric disorders. It is also frequently intertwined with factors like family support quality, caregiving stability, and stress coping styles, making patients more prone to developing and maintaining depressive symptoms against the backdrop of chronic OCD symptoms [24, 25]. Concurrently, early-life stress related to family history can shape more rigid cognitive schemas, leading to a greater tendency towards persistent negative interpretations and self-blame when encountering repeated intrusions from OCD symptoms [26, 27]. This finding suggests that in clinical assessment, family history should be considered crucial information for stratified management, and early mood screening and family support interventions are necessary for populations with high genetic vulnerability. However, a large-scale genetic study by Grotzinger *et al*. [28] on 11 major psychiatric disorders, using genomic structural equation modelling, identified four major genetic factors (neurodevelopmental, compulsive, psychotic, internalizing). While finding moderate to high genetic correlations between disorders, they noted the limited utility of single-dimensional genetic risk at the level of biobehavioural associations or individual variation. This finding suggests that the comorbidity risk between OCD and depression may not derive from a straightforward shared genetic background, but from complex interactions of multidimensional genetic factors. Patients with a positive family history might carry heavy loads of internalizing and compulsive factors, which could reduce the threshold for the expression of dual pathology [29, 30, 31].

Multivariate regression analysis results showed that comorbid anxiety, family history of psychosis, and moderate-severe obsessive-compulsive symptoms were risk factors for comorbid depression in patients with OCD. Furthermore, total Y-BOCS score showed a significant positive correlation with HAMD score. This phenomenon may reflect that the overt functional impairment caused by compulsive behaviours more directly affects life rhythms and social roles, such as time consumption, work/study impairment and interpersonal avoidance, thereby promoting worsening depression through tangible losses and reduced social support [32]. By contrast, persistent intrusive obsessive thoughts readily induce psychological distress and increased ruminative processing, fostering the maintenance of depression through negative self-evaluation and impaired emotional regulation. Under the combined influence of both, the total burden of obsessive-compulsive symptoms and depression severity tend to rise synchronously. This finding suggests that in clinical follow-up, changes in Y-BOCS scores can serve as dynamic indicators of emotional risk, and attention should also be paid to the degree of functional erosion caused by compulsive behaviours to enhance the sensitivity of depression risk identification [25].

However, while the univariate analysis observed a higher proportion of out-of-pocket payment in the depression group, it did not reach statistical significance in the multivariate model. This result could be influenced by sample size limitations and interactions between variables. It may also reflect that payment type more broadly represents economic pressure and differences in healthcare accessibility, with its effect on depression being indirect: economic burden can exert influence by increasing stress, reducing treatment continuity, and weakening social support. Its independent effect weakens once more proximal factors such as OCD severity and comorbid anxiety are incorporated into the model. The clinical implication of this finding is that economic pressure is more suitable as one component of a comprehensive assessment and needs to be interpreted in conjunction with treatment adherence, medication availability, and social support.

The value of this study lies in its use of retrospective case data, combining LASSO screening and logistic regression to construct a framework of risk factors, and validating the linear correlation between OCD symptom burden and depression burden in a comorbid depression population. This provides an operational set of indicators for stratified identification of depression comorbidity in OCD. However, limitations exist. As a single-centre retrospective study, it cannot determine the temporal sequence between OCD and depressive symptoms. Future research should adopt multicentre prospective cohort designs, with repeated assessments of Y-BOCS and HAMD-17 at admission and at 4, 8, and 12 weeks and 6-month follow-ups to characterize dynamic symptom trajectories. Furthermore, key confounding factors such as treatment modalities, medication dosage, psychotherapy exposure, adherence, social support, and quality of life were not systematically included. Future studies should establish standardized case report forms to record medication types, dosage modifications, number of exposure and response prevention sessions, reasons for follow-up interruption, and family support levels. Moreover, the model was not externally validated; future work should expand sample sources, use training and validation sets for modelling, and employ calibration curves, decision curve analysis, and resampling internal validation to assess model stability.

## Conclusions

This case-control study identified comorbid anxiety, a family history of psychosis, and greater severity of OCD as independent risk factors for comorbid depression in patients with OCD. Y-BOCS total score, obsession score, and compulsion score were all positively correlated with HAMD-17 scores, with the compulsion score showing the highest correlation. These findings suggest that comorbid anxiety, family history, and obsessive-compulsive symptom dimensions can function as clinically accessible indicators for screening and stratified management of comorbid depression in individuals with OCD. Future research should adopt multicentre prospective cohort designs to characterize dynamic symptom trajectories and include key confounding factors such as treatment modalities, medication adherence, and social support to further validate and refine the risk model.

## Availability of Data and Materials

The clinical data that support the findings of this study are available from the corresponding author upon reasonable request.

## References

[1] Brock H, Rizvi A, Hany M (2026). Obsessive-compulsive disorder. https://www.ncbi.nlm.nih.gov/books/NBK553162/.

[2] Virtanen S, Kuja-Halkola R, Sidorchuk A, Fernández de la Cruz L, Rück C, Lundström S (2022). Association of Obsessive-Compulsive Disorder and Obsessive-Compulsive Symptoms With Substance Misuse in 2 Longitudinal Cohorts in Sweden. *JAMA Network Open*.

[3] Wang YL, Wang JR, Huang BL, Yang XH, Guo HR, Ren YM (2022). Changes in the psychological defense mechanism and clinical features of patients with OCD - A four-year follow-up study. *Journal of Affective Disorders*.

[4] Bramante S, Maina G, Borgogno R, Pellegrini L, Rigardetto S, Albert U (2023). Assessing suicide risk in patients with obsessive-compulsive disorder: a dimensional approach. *Revista Brasileira de Psiquiatria: Orgão Oficial da Associação Brasileira de Psiquiatria, Asociación Psiquiátrica de la América Latina*.

[5] Mavrogiorgou P, Becker S, Lee-Grimm SI, Juckel G (2023). Embitterment and metacognition in obsessive-compulsive disorder. *BMC Psychiatry*.

[6] Battle DE (2013). Diagnostic and Statistical Manual of Mental Disorders (DSM). *CoDAS*.

[7] Zimmerman M, Martinez JH, Young D, Chelminski I, Dalrymple K (2013). Severity classification on the Hamilton depression rating scale. *Journal of Affective Disorders*.

[8] Chinese Medical Association (2016). Chinese guidelines for the prevention and treatment of obsessive-compulsive disorder 2016 (abridged edition). *Chinese Journal of Psychiatry*.

[9] Hamilton M (1960). A rating scale for depression. *Journal of Neurology, Neurosurgery, and Psychiatry*.

[10] Goodman WK, Price LH, Rasmussen SA, Mazure C, Fleischmann RL, Hill CL (1989). The Yale-Brown Obsessive Compulsive Scale. I. Development, use, and reliability. *Archives of General Psychiatry*.

[11] Superior HealthPlan (2021). Deep transcranial magnetic stimulation for the treatment of obsessive compulsive disorder. https://www.superiorhealthplan.com/content/dam/centene/Superior/Provider/clinical-policies/CP.BH.201.pdf.

[12] Faries D, Herrera J, Rayamajhi J, DeBrota D, Demitrack M, Potter WZ (2000). The responsiveness of the Hamilton Depression Rating Scale. *Journal of Psychiatric Research*.

[13] Pan W, Liu P, He M, Sun M, Xiao Y, Wang Z (2025). Associations between obsessive-compulsive symptoms, impulsivity, and suicidality among Chinese college students: a latent profile analysis and mediation analysis. *BMC Psychology*.

[14] Perris F, Cipolla S, Catapano P, Sampogna G, Luciano M, Giallonardo V (2023). Duration of Untreated Illness in Patients with Obsessive-Compulsive Disorder and Its Impact on Long-Term Outcome: A Systematic Review. *Journal of Personalized Medicine*.

[15] Friedman-Ezra A, Keydar-Cohen K, van Oppen P, Eikelenboom M, Schruers K, Anholt GE (2024). Loneliness in OCD and its determinants. *Psychiatry Research*.

[16] Mishra V, Suthar N, Swami MK (2025). Association of Depression with Phenomenology and Insight Among Patients with Obsessive-Compulsive Disorder. *Nigerian Medical Journal: Journal of the Nigeria Medical Association*.

[17] Moritz S, Von Mühlenen A, Randjbar S, Fricke S, Jelinek L (2009). Evidence for an attentional bias for washing- and checking-relevant stimuli in obsessive-compulsive disorder. *Journal of the International Neuropsychological Society: JINS*.

[18] Choi SA, Lee JH (2015). The effect of enhanced responsibility on attentional bias in obsessive-compulsive checkers. *Journal of Obsessive-Compulsive and Related Disorders*.

[19] Ravi M, Miller AH, Michopoulos V (2021). The immunology of stress and the impact of inflammation on the brain and behaviour. *BJPsych Advances*.

[20] Hole C, Dhamsania A, Brown C, Ryznar R (2025). Immune dysregulation in depression and anxiety: a review of the immune response in disease and treatment. *Cells*.

[21] Nguyen VV, Zainal NH, Newman MG (2022). Why Sleep is Key: Poor Sleep Quality is a Mechanism for the Bidirectional Relationship between Major Depressive Disorder and Generalized Anxiety Disorder Across 18 Years. *Journal of Anxiety Disorders*.

[22] Belli GM, Law C, Mancebo M, Eisen J, Rasmussen S, Boisseau CL (2023). Directionality of change in obsessive compulsive disorder and depression over six years of prospective follow-up. *Journal of Psychiatric Research*.

[23] Hathway T, McDonald S, Melkonian M, Karin E, Titov N, Dear BF (2024). Correlates of depression in individuals with obsessive compulsive disorder. *Cognitive behaviour therapy*.

[24] Hsu TW, Tsai SJ, Bai YM, Cheng CM, Su TP, Chen TJ (2024). Parental mental disorders in patients with comorbid schizophrenia and obsessive-compulsive disorder: a nationwide family-link study. *European Child & Adolescent Psychiatry*.

[25] Kendler KS, Ohlsson H, Sundquist J, Sundquist K (2022). Risk for Mood, Anxiety, and Psychotic Disorders in Individuals at High and Low Genetic Liability for Bipolar Disorder and Major Depression. *JAMA Psychiatry*.

[26] Cabana-Domínguez J, Torrico B, Reif A, Fernàndez-Castillo N, Cormand B (2022). Comprehensive exploration of the genetic contribution of the dopaminergic and serotonergic pathways to psychiatric disorders. *Translational psychiatry*.

[27] Uher R, Pavlova B, Radua J, Provenzani U, Najafi S, Fortea L (2023). Transdiagnostic risk of mental disorders in offspring of affected parents: a meta-analysis of family high-risk and registry studies. *World Psychiatry: Official Journal of the World Psychiatric Association (WPA)*.

[28] Grotzinger AD, Mallard TT, Akingbuwa WA, Ip HF, Adams MJ, Lewis CM (2022). Genetic architecture of 11 major psychiatric disorders at biobehavioral, functional genomic and molecular genetic levels of analysis. *Nature Genetics*.

[29] Hinduja S, Darzi A, Ertugrul IO, Provenza N, Gadot R, Storch EA (2024). Multimodal Prediction of Obsessive-Compulsive Disorder and Comorbid Depression Severity and Energy Delivered by Deep Brain Electrodes. *IEEE Transactions on Affective Computing*.

[30] Samuels J, Bienvenu OJ, Krasnow J, Grados MA, Cullen BA, Goes FS (2023). Prevalence and correlates of lifetime suicide attempt in obsessive-compulsive disorder with major depression. *Journal of Psychiatric Research*.

[31] Bolhuis K, McAdams TA, Monzani B, Gregory AM, Mataix-Cols D, Stringaris A (2014). Aetiological overlap between obsessive–compulsive and depressive symptoms: a longitudinal twin study in adolescents and adults. *Psychological Medicine*.

[32] Belli GM, Law C, Obisie-Orlu IC, Eisen JL, Rasmussen SA, Boisseau CL (2024). Course and clinical correlates of obsessive-compulsive disorder with or without comorbid personality disorder. *Journal of Affective Disorders*.

